# LTB4R Promotes the Occurrence and Progression of Clear Cell Renal Cell Carcinoma (ccRCC) by Regulating the AKT/mTOR Signaling Pathway

**DOI:** 10.3390/cells11223606

**Published:** 2022-11-15

**Authors:** Xiao Zhang, Huahui Wu, Xin Yan, Jiajun Ma, Zhao Chen

**Affiliations:** 1 Department of Urology, Zhongnan Hospital of Wuhan University, Wuhan 430071, China; 2 Department of Emergency, Yiwu Traditional Chinese Medicine Hospital, 266, Xuefeng West Road, Yiwu 322000, China; 3 Department of Cardiovascular Surgery, Zhongnan Hospital of Wuhan University, Wuhan 430071, China

**Keywords:** LTB4R, clear cell renal cell carcinoma, AKT/mTOR signaling pathway, apoptosis, cell cycle

## Abstract

ccRCC is highly immunogenic, yet its underlying immune-related molecular mechanisms are unknown. Leukotriene B4 Receptor 1 (LTB4R), a novel immune-related gene associated in our previous research with the prognosis of ccRCC patients, has been found in many cancers; however, its potential mechanism in renal clear carcinoma is unclear. This study was conducted to investigate LTB4R’s action mechanism in renal clear cell carcinoma. First, a CCK8 assay was performed to verify LTB4R’s effect on the proliferation viability of renal clear cell carcinoma cells. Scratch and transwell assays verified LTB4R’s effect on the migration and invasion ability of renal clear cell carcinoma cells. Flow cytometry validated LTB4R’s effect on renal clear cell carcinoma cells’ apoptosis and cell cycle. A Western blot assay was conducted to further investigate LTB4R’s effect on apoptosis, cell cycle, EMT process, and AKT/mTOR signaling pathway in renal clear cell carcinoma at the protein level. In vitro experiments showed that LTB4R knockdown inhibited the proliferation, migration, and invasion of renal clear cell carcinoma cells and promoted their apoptosis, whereas LTB4R overexpression promoted the proliferation, migration, and invasion of renal clear cell carcinoma cells and inhibited their apoptosis. In addition, we found that LTB4R regulated the proliferation and apoptosis of renal clear cell carcinoma cells by regulating the AKT/mTOR signaling pathway’s phosphorylation process. Furthermore, we verified some of these results using bioinformatic analysis. LTB4R plays an oncogenic role in renal clear cell carcinoma; it is expected to be a molecular target for renal clear cell carcinoma treatment and a predictive biomarker for prognosis.

## 1. Introduction

Renal cell carcinoma (RCC) is one of the most common tumors in the urinary system, and is derived from renal tubular epithelial cells [1]; it will result in an estimated 79,000 new cases and 13,920 deaths in 2022 [2]. As the most common pathological RCC type, clear cell renal cell carcinoma (ccRCC) accounts for approximately eighty-five percent of RCC cases and has a poorer prognosis compared to papillary and suspicious renal cell carcinoma [3]. However, ccRCC is insensitive to both radiotherapy and chemotherapy [4,5].

Nowadays, a growing number of studies have shown that immunotherapy can effectively and safely treat tumors [6,7,8]. Recent studies have also shown that immune-associated genes (IAG) play an important role in the development and progression of renal clear cell carcinoma [9,10].

The combination of leukotriene B4 receptor 1 (LTB4R) with LTB4 to activate leukocytes and prolong their survival as IAGs is significant in acute inflammatory responses [11,12,13]. BLT1 (LTB4R) is also an attractive drug target for allergic airway inflammation, inflammatory arthritis, atherosclerosis, and psoriasis [11]. However, BLT1 expression is critical for CD8+ cell infiltration into tumors and MMP-2 and IL-8 pathways, which increases the invasiveness of ovarian and breast cancer cells [14,15,16]. In summary, recent studies highlight BLT1’s critical role in diseases such as inflammation and tumors, as well as its potential as a therapeutic target for certain cancers.

AKT is a key factor in regulating the functions of various substrates in cell survival and growth [17]. A large body of work supports the idea that AKT is an important therapeutic target. However, all other AKT pathway components, including PI3Ks, PDK1, AKT, and various isoforms of mTOR, are also the focus of drug development and molecularly targeted cancer therapies [18]. A recent study reported that LTB4 directly promoted myofibroblast differentiation from fibroblasts and endothelial cells via BLT1 and subsequent PI3K/AKT/mTOR signaling [19].

Therefore, this study aims to investigate the relationship between BLT1 and the AKT pathway in renal clear cell carcinoma as well as BLT1’s biological function in the development and progression of renal clear cell carcinoma and its action mechanism. Doing so will provide a reliable theoretical and experimental basis for the prevention and treatment of renal clear cell carcinoma, in the hope of finding new therapeutic targets for ccRCC.

## 2. Materials and Methods

### 2.1. ccRCC Cohort Collection and Preprocessing

First, the ccRCC expression cohort data were retrieved from The Cancer Genome Atlas (TCGA) and Gene Expression Omnibus (GEO) databases [20,21]. A total of 530 ccRCC samples and 129 normal samples were obtained from these databases (TCGA-KIRC cohort). Another 39 normal samples were obtained from the Genotype-Tissue Expression (GTEx) database for subsequent analysis [22]. Related clinical information, including living status, survival time, and clinicopathological stage, was also obtained to explore the association of LTB4R expression with clinical features and prognostic value. TCGA and GTEx databases were used to compare differences in LTB4R expression between ccRCC samples and normal samples. An independent dataset, GSE53757 (including 72 ccRCC samples), was included for Spearman correlation analysis.

### 2.2. Cell Recovery and Culture

ACHN and Caki1 cells were obtained from the Chinese Academy of Medical Sciences (Beijing, China). Frozen cells were thawed in a 37° water bath, centrifuged to remove the supernatant, mixed with culture medium, transferred to a cell culture flask, and incubated in a cell culture incubator. ACHN and Caki1 cells were cultured in MEM (Gibco, Waltham, MA, USA) and McCoy’s 5A (Gibco, Waltham, MA, USA) mediums including 10% FBS (Gibco, Waltham, MA, USA), respectively.

### 2.3. Plasmid and siRNA Transfection

LTB4R plasmid and the control plasmid were designed and synthesized by FengHui Biology Company (Wuhan, China). The siRNAs and control RNA were purchased from GenePharma (Shanghai, China). The LTB4R corresponding sense sequences were as follows: Si-LTB4R*1: 5′-GCUUCCCGGCAACAGCUUUTT-3′, 3′-AAAGCUGUUGCCGGGAAGCTT-5′; Si-LTB4R*2: 5′-CCCAGAAGCUACGCACCAATT-3′, 3′-UUGGUGCGUAGCUUCUGGGTT-5′. GP-transfect-Mate (GenePharma, Shanghai, China) was used for cell transfection.

### 2.4. RNA Extraction and Real-Time Quantitative PCR Detection (qRT-PCR)

RNA was extracted using TRIzol Reagent (T9108, Takara, Dalian, China) and reverse transcription was performed using an enzyme kit (RK21203, ABclonal, Wuhan, China). Finally, cDNA was analysed via qRT-PCR using Green Supermix (Bio-RAD, Hercules, CA, USA). Primer sequences were as follows: LTB4R: 5′-AGCTTTGTGGTGTGGAGTATCC-3′, 3′-GCAACCAGCCAGTCCAAAAC-5′ and GAPDH: 5′-GGAGCGAGATCCCTCCAAAAT-3′, 3′-GGCTGTTGTCATACTTCTCATGG-5′.

### 2.5. CCK8 Assays

Cells were trypsinized and mixed for counting. Next, 3000 cells were seeded into 96-well plates and set up as 0 h, 24 h, and 48 h groups, with three replicates in each group. Cells adhered for 4 h, then 10 μL of CCK-8 reagent (C0037, Beyotime Biotechnology, Jiangsu, China) was added in the dark, and cells were returned to the incubator for 2 h. A microplate reader detected the absorbance value (450 nm). Following this, the absorbance value was measured every 24 h, at 24 h, 48 h, and 72 h. Finally, GraphPad Prism8 was used to make a cell proliferation curve.

### 2.6. Colony Formation Assays

Cells were trypsinized, mixed with medium, and then counted. Approximately 200 cells were seeded into 6-well plates and incubated for 2 weeks. When clumps were visible in the 6-well plates, the medium was aspirated, washed three times with PBS, and fixed with 4% formaldehyde for 15 min. After aspirating the formaldehyde, the 6-well plates were stained using 0.1% crystal violet for 30 min, washed with PBS, blown dry, and photographed; then clones were counted.

### 2.7. Transwell Experiment

After trypsin digested the cells, the supernatant was removed using centrifugation and cells were re-suspended using a culture medium. Cell density was adjusted to 105/mL, 200 μL of serum-free medium was added to the chamber, and 500 μL of serum-containing medium was added outside the chamber. After 24 h, the chamber was fixed in 4% formaldehyde for 15 min, stained using 0.1% crystal violet solution for 30 min, washed with PBS, and placed under an inverted microscope for observation and photography.

### 2.8. Flow Cytometry for Apoptosis and Cell Cycle

After trypsin digestion, cells were collected for analysis and 1× binding buffer was added; then cells were re-suspended and centrifuged at 300× *g*/min for 10 min to remove the supernatant. Next, 5 μL Annexin V-FITC (556420, BD Biosciences, Franklin Lakes, NJ) and 10 μL PI (550825, BD Biosciences) were added; the suspension was mixed well and incubated in the dark at room temperature. After 10 min, results were analyzed on a machine for apoptosis. Next, cells collected using the method noted above in Section 2.1, were re-suspended in 1× binding buffer; the suspension was aspirated; 1 mL of DNA staining solution (BD Biosciences) and 10 μL of permeabilization solution (554722, BD Biosciences) were added; the suspension was incubated for 30 min in the dark; and then the cell cycle was analyzed using a machine.

### 2.9. Western Blotting

Total protein was extracted using RIPA buffer (R0010, Solarbio, Beijing, China). Protein extracts were transferred to PVDF membranes, and then incubated using appropriate antibodies. An ECL imaging system (Tanon-5200, Shanghai, China) was used to detect ECL signals. The primary antibodies used were: anti-LTB4R (ERP7113, 1:1000, Abcam, Carlsbard, CA, USA); anti-GAPDH (A19056, 1:1000, ABclonal, Hubei, China), anti-CDK2 (A0094, 1:1000, ABclonal), anti-CDK4 (A11136, 1:1000, ABclonal), anti-cyclin D1 (A19038, 1:1000, ABclonal), anti-Bcl-2 (A19693, 1:1000, ABclonal), anti-Bax (A19684, 1:1000, ABclonal), anti-caspase3 (9662, 1:1000, Cell Signaling Technology), anti-cleaved-caspase3 (Asp175, 1:1000, Cell Signaling Technology), anti-vimentin (A19607, 1:1000, ABclonal), anti-NCA (A19083, 1:1000, ABclonal), anti-ECA (A18135, 1:1000, ABclonal), anti-AKT (A17909, 1:1000, ABclonal), anti-P-AKT (AP0637, 1:1000, ABclonal), anti-mTOR (A11355, 1:1000, ABclonal), and anti-P-mTOR (AP0115, 1:1000, ABclonal). The second antibody was Anti-Rabbit-IgG(H+L)-HRP (AS030, 1:10,000, ABclonal).

### 2.10. Statistical Analysis

Data were analyzed using Prism 8.0 (GraphPad Software, La Jolla, CA, USA) and SPSS 26.0 (IBM SPSS Statistics for Windows, version 26.0). All data are presented as mean ± SD (standard deviation). Data analysis was performed using Student’s *t*-test, χ^2^ test, Fisher’s exact test, etc. *p* < 0.05 was considered to indicate a significant difference.

## 3. Results

### 3.1. LTB4R Expression and Its Effect on Survival and Clinical Stages in ccRCC

**Figure 1:**
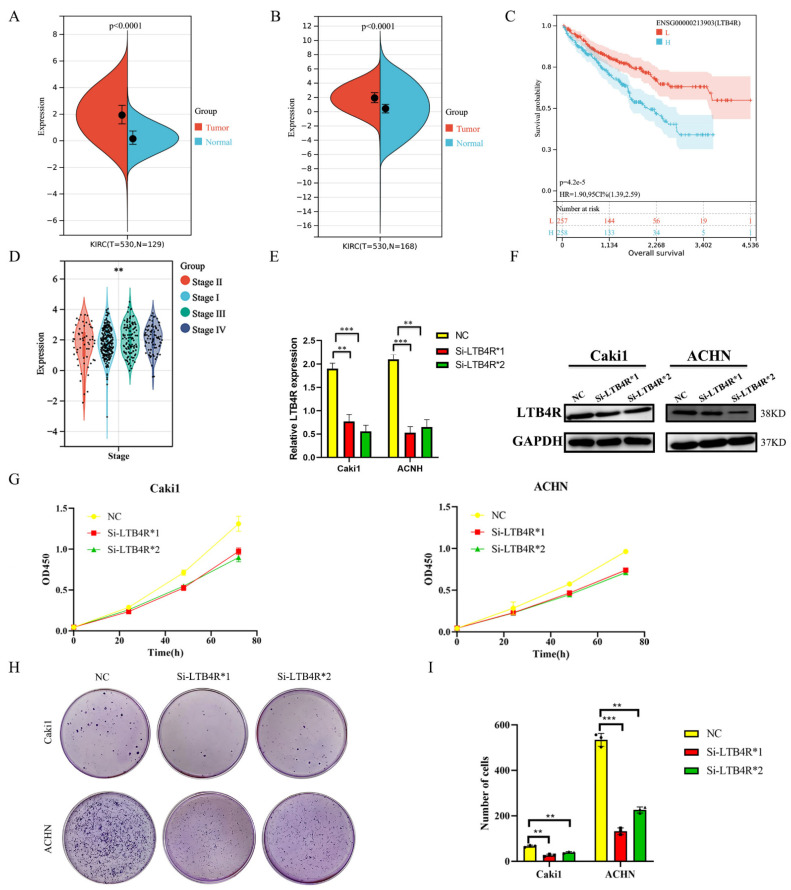
The expression of LTB4R and its relationship with survival and clinical traits in ccRCC. LTB4R knockdown inhibited the proliferation and colony formation of ccRCC cells. (**A**) Expression difference of LTB4R between ccRCC samples (*n* = 530) and normal samples (*n* = 129) by using TCGA and GTEx databases. Wilcoxon rank-sum test, *p* < 0.0001. (**B**) Expression difference of LTB4R between ccRCC samples (*n* = 530) and normal samples (*n* = 168) by using TCGA database. Wilcoxon rank-sum test, *p* < 0.0001. (**C**) Risk model survival curve analysis. Log-rank test, *p* = 4.2 × 10^−5^. (**D**) High expression of LTB4R was related to higher tumor stage. Wilcoxon rank-sum test, ** *p* < 0.001. (**E**) RT-qPCR detected the knockdown efficiency of LTB4R. (**F**) Western Blot Analysis to verify the knockdown efficiency of LTB4R. (**G**) Knockdown of LTB4R inhibited the proliferation of Caki1 and ACHN cells. (**H**,**I**) After knockdown of LTB4R, the cloning formation ability of Caki1 and ACHN cells were decreased. A 2-tailed *t* test was used in E and I (** *p <* 0.01, *** *p <* 0.001).

LTB4R was over expressed in ccRCC samples (*n* = 530) compared to normal samples (*n* = 129) in the TCGA and GTEx databases (Figure 1A, *p* < 0.0001); this was further verified using TCGA-KIRC data (Figure 1B, *p* < 0.0001). In addition, we explored differences in clinical stages among LTB4R expressions (Figure 1D). In the TCGA-KIRC cohort, patients with a higher LTB4R expression showed a higher clinical stage (*p* < 0.01). Survival data from the TCGA database suggested that patients with higher LTB4R expressions showed significantly poorer overall survival (OS) than patients with lower LTB4R expressions (*p* = 4.2 × 10^−5^, HR = 1.9, 95 CI% (1.39, 2.59), Figure 1C).

### 3.2. Interfering with LTB4R Inhibited the Proliferation and Clone Formation of Renal Cancer Cells In Vitro

We designed siRNAs (Si-LTB4R*1 and Si-LTB4R*2) and a blank control (Si-NC) to transfect Caki1 and ACHN cells. RT-qPCR and Western blotting validated the efficiency of siRNAs LTB4R knockdown (Figure 1E,F). After knocking down LTB4R, we found that the proliferation and clone-forming ability of Caki1 and ACHN cells were significantly increased compared with the control group using CCK8 and colony formation experiments (Figure 1G–I).

### 3.3. LTB4R Knockdown Promoted Apoptosis and Inhibited Cell Cycle Progression in ccRCC Cells

**Figure 2:**
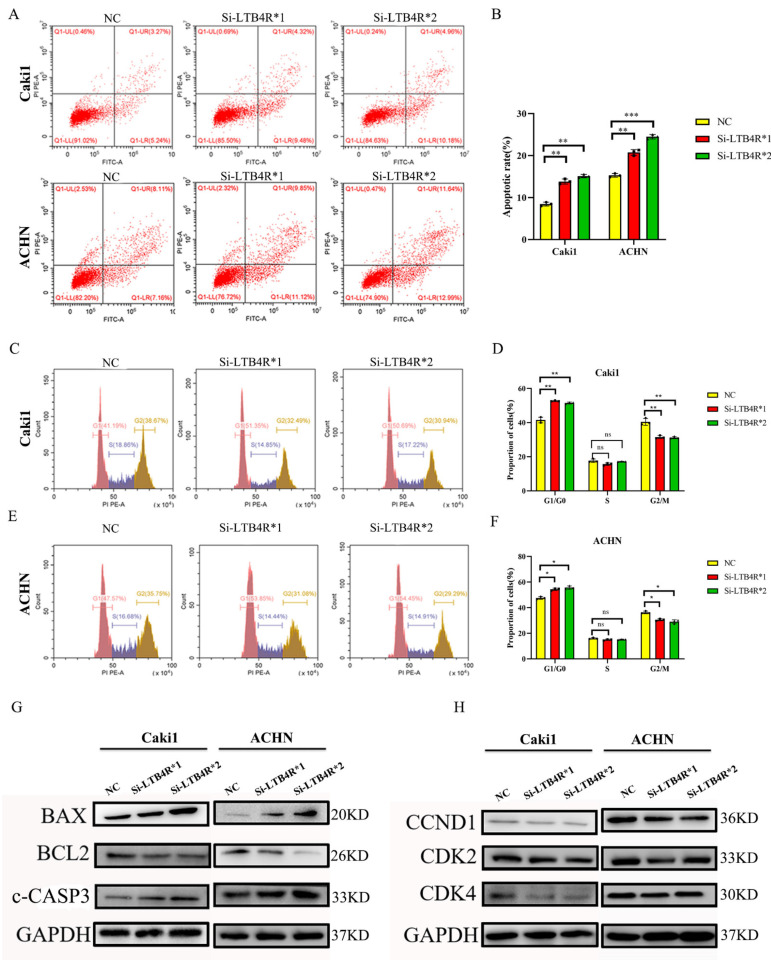
Interfering with LTB4R inhibited ccRCC cell cycle and promoted ccRCC cell apoptosis. (**A**,**B**) Knockdown of LTB4R promoted apoptosis in Caki1 and ACHN cells. (**C**–**F**) After knockdown of LTB4R, the proportion of the G1/G0 phase in Caki1 and ACHN cells increased, while the proportion of the G2/M phase decreased, and the proportion of the S phase did not change significantly. (**G**) Western Blot Analysis showed that after knockdown of LTB4R, the apoptosis-related proteins, BAX and c-CASP3 (c-caspase-3) were increased and the anti-apoptotic protein BCL2 was decreased in Caki1 and ACHN cells. (**H**) Western Blot Analysis showed that the period-related proteins CCND1 (CyclinD1), CDK2, and CDK4 were all reduced after LTB4R knockdown. A 2-tailed *t* test was used (* *p <* 0.05; ** *p* < 0.01, *** *p* < 0.001, ns, not significant).

We assessed the influence of LTB4R knockdown on cell apoptosis and the cell cycle using flow cytometry. The results showed that interfering with LTB4R could promote cell apoptosis (Figure 2A,B) and block ccRCC cells’ cell cycle in the G1/GO phase (Figure 2C–F). In addition, levels of apoptosis proteins (Bax and c-CASP3) significantly increased, BCL2 decreased (Figure 2G), and levels of periodic proteins (CCND1, CDK2, and CDK4) dramatically decreased (Figure 2H).

### 3.4. LTB4R Knockdown Suppressed Migration, Invasion, and Epithelial-Mesenchymal Transformation (EMT)

**Figure 3:**
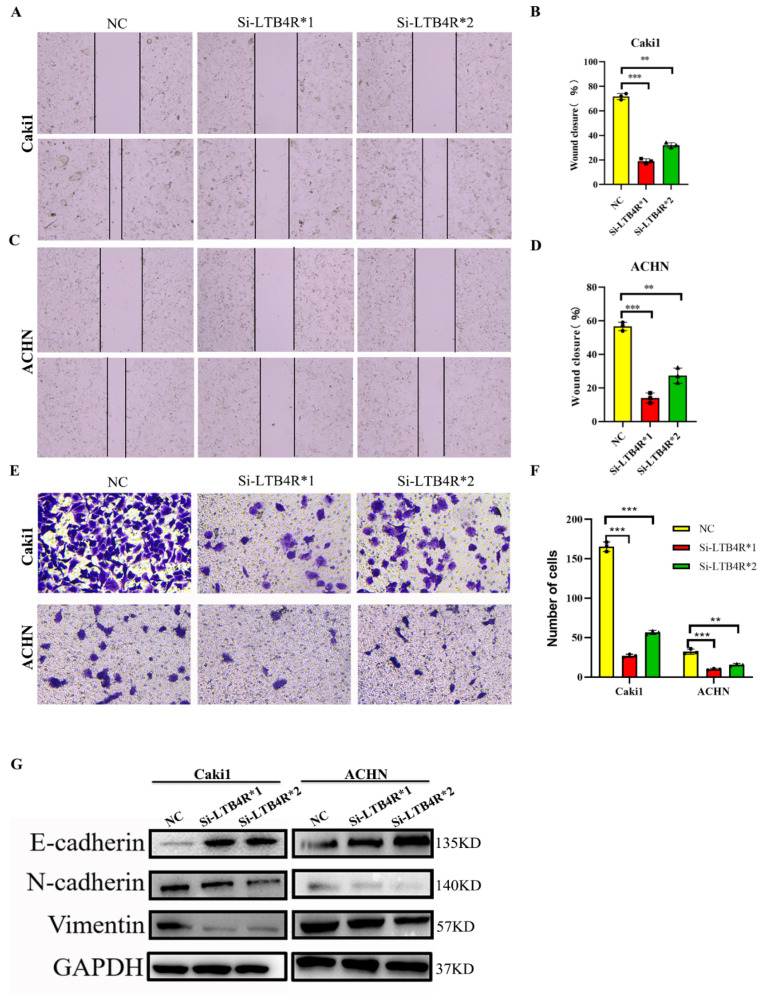
Interfering with LTB4R inhibited the ability of ccRCC cells to migrate and invade, and EMT progression. (**A**–**D**) Knockdown of LTB4R inhibited the migration of Caki1 cells and ACHN cells. (**E**,**F**) Knockdown of LTB4R inhibited the invasion of Caki1 and ACHN cells. (**G**) Western blot analysis showed that knockdown of LTB4R increased the epithelial phenotype E-cadherin and significantly decreased the mesenchymal phenotype (N-cadherin, Vimentin) in Caki1 and ACHN cells. A 2-tailed *t* test was used (** *p* < 0.01, *** *p* < 0.001).

First, scratch experiments indicated decreased migratory abilities, and transwell assays displayed decreased invasive abilities of Caki1 and ACHN cells transfected Si-LTB4R compared with the control group (Figure 3A–F). In contrast, some studies reported that EMT was related to tumorigenesis, progression, and recurrence [23,24]. We speculated that LTB4R promoted the EMT process, and Western blot results confirmed this; N-cadherin and vimentin levels significantly decreased in Caki1 and ACHN cells after LTB4R knockdown, whereas E-cadherin levels dramatically increased (Figure 3G).

### 3.5. LTB4R Promoted the Proliferation and Clone Formation of ccRCC Cells

**Figure 4:**
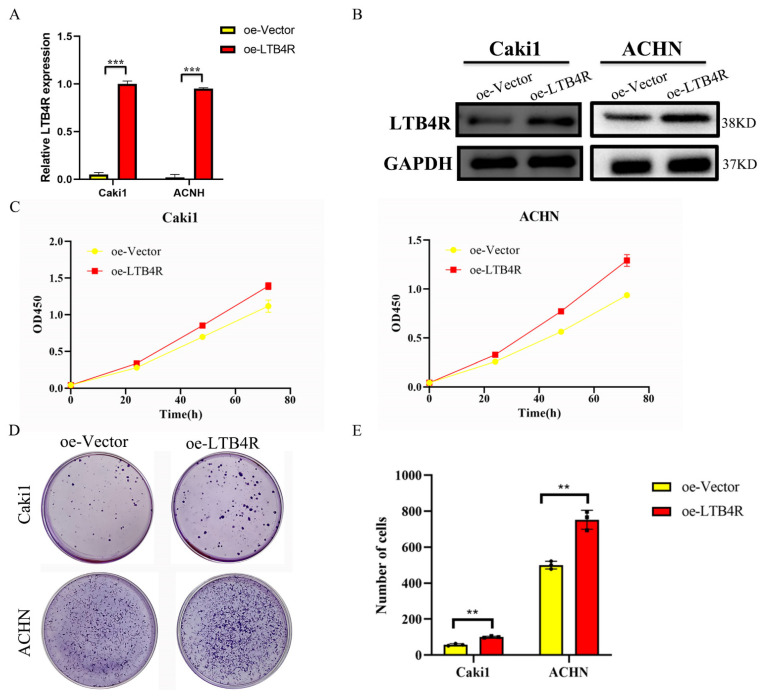
Overexpression of LTB4R promoted proliferation and clone formation of ccRCC cells. (**A**) RT-qPCR detection of LTB4R overexpression efficiency. (**B**) Western blot experiments to verify the overexpression efficiency of LTB4R. (**C**) Overexpression of LTB4R promoted the proliferative capacity of Caki1 and ACHN cells. (**D**,**E**) Overexpression of LTB4R promoted clone formation of Caki1 and ACHN cells. A 2-tailed *t* test was used (** *p <* 0.01, *** *p <* 0.001).

After the construction of plasmids (oe-Vector and oe-LTB4R), we used plasmids to transfect Caki1 and ACHN cells. RT-qPCR and Western blotting validated the efficiency of plasmids’ overexpression of LTB4R (Figure 4A,B). CCK8 assay and clone assay results indicated that the proliferation and clone-forming abilities of Caki1 and ACHN cells were significantly increased compared to the control group after LTB4R was overexpressed (Figure 4C–E).

### 3.6. LTB4R Suppressed Apoptosis and Promoted Cell Cycle Progression in ccRCC Cells

**Figure 5:**
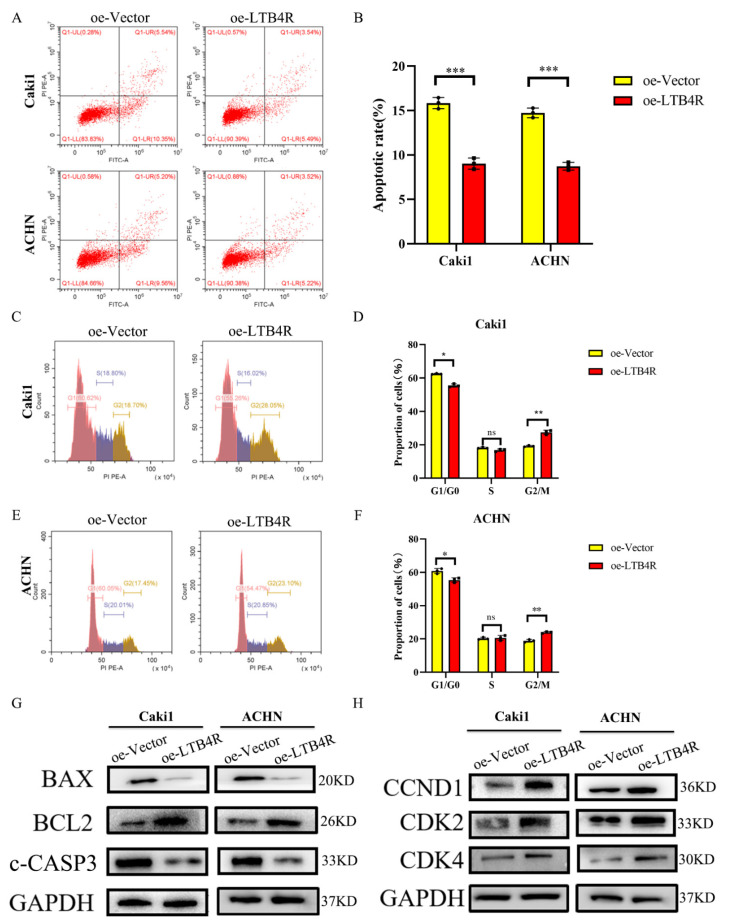
LTB4R overexpression promoted the cell period and inhibited apoptosis of ccRCC cells. (**A**,**B**) LTB4R overexpression inhibited apoptosis of Caki1 and ACHN cells, respectively. (**C**–**F**) After LTB4R overexpression, the proportion of G1/G0 phase in Caki1 and ACHN cells decreased, whereas the proportion of G2/M phase increased, and the proportion of S phase did not significantly change. (**G**) Western blot analysis showed that after LTB4R overexpression, the expression of apoptosis-related proteins BAX and c-CASP3 (c-caspase-3) decreased, and the expression of anti-apoptotic protein BCL2 increased. (**H**) Western blot analysis showed that the expression of period-related proteins CCND1 (cyclin D1), CDK2, and CDK4 increased after LTB4R overexpression. A 2-tailed *t* test was used (* *p <* 0.05; ** *p* < 0.01; *** *p* < 0.001; and ns, not significant).

Flow cytometry results showed that LTB4R overexpression could suppress cell apoptosis and promote the cell cycle in ccRCC cells (Figure 5A–F). Other than that, levels of apoptosis proteins (Bax and c-CASP3) significantly decreased and BCL2 increased (Figure 5G), whereas levels of the periodic proteins (CCND1, CDK2, and CDK4) dramatically increased after LTB4R was overexpressed (Figure 5H).

### 3.7. LTB4R Promoted Migration, Invasion, and Epithelial–Mesenchymal Transformation (EMT)

**Figure 6:**
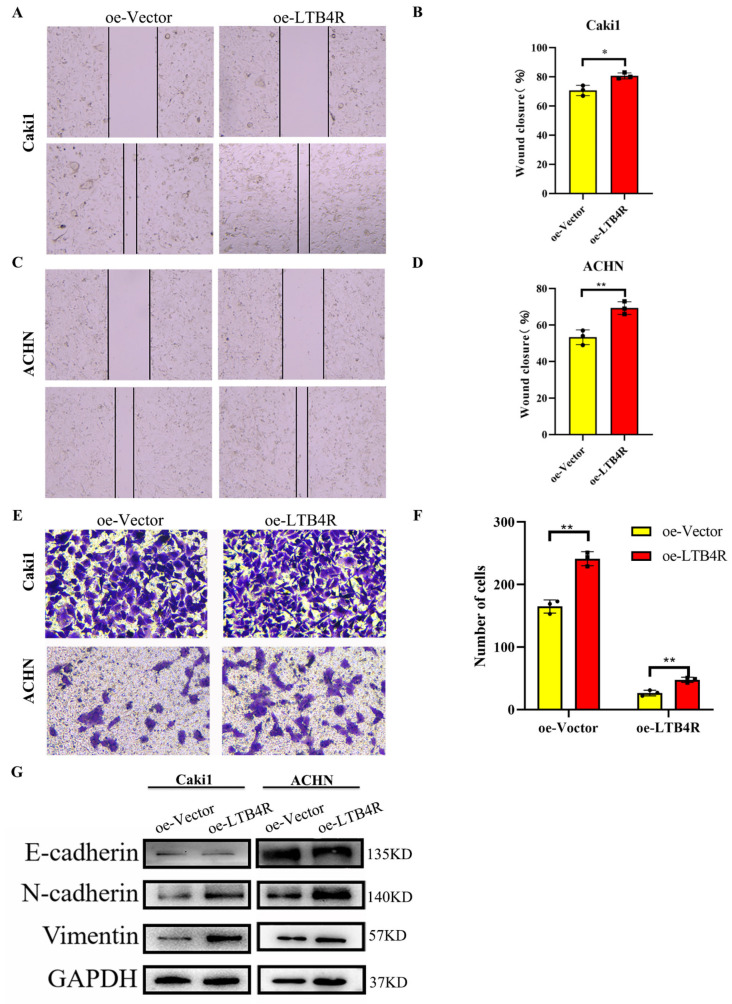
Overexpression of LTB4R promoted the migration, invasion and EMT process of ccRCC cells. (**A**–**D**) Overexpression of LTB4R promoted the migratory ability of Caki1 and ACHN cells. (**E**,**F**) Overexpression of LTB4R promoted the clonogenicity of Caki1 and ACHN cells. (**G**) Western blot Analysis showed that overexpression of LTB4R significantly decreased the epithelial phenotype E-cadherin and increased mesenchymal phenotype (N-cadherin, Vimentin) of Caki1 and ACHN cells. A 2-tailed *t* test was used (* *p* < 0.05, ** *p* < 0.01).

After LTB4R overexpression, we conducted scratch experiments, transwell assays, and Western blotting. Scratch experiments’ results showed increased migratory abilities (Figure 6A–D) and transwell assays displayed Caki1 and ACHN cells increased invasive abilities (Figure 6E,F) compared with the control group. Western blot results showed that N-cadherin and vimentin levels significantly increased, whereas E-cadherin dramatically decreased in Caki1 and ACHN cells (Figure 6G).

### 3.8. LTB4R Expression Was Related to Apoptosis, Cell Cycle, and EMT

**Figure 7:**
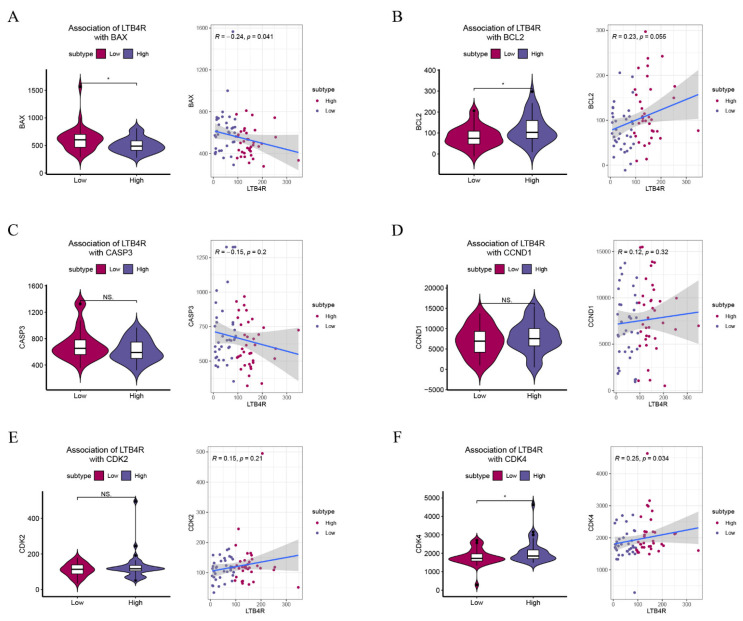
The association of LTB4R with BAX, BCL2, CASP3, CCND1, CDK2, and CDK4. (**A**–**F**) The difference of BAX, BCL2, CASP3, CCND1, CDK2, and CDK4 scores defined by using ssGSEA between LTB4R high expressed group and LTB4R low expressed group. In addition, the association of LTB4R expression with BAX, BCL2, CASP3, CCND1, CDK2, and CDK4 scores. Wilcoxon rank-sum test, * *p* < 0.05.

**Figure 8:**
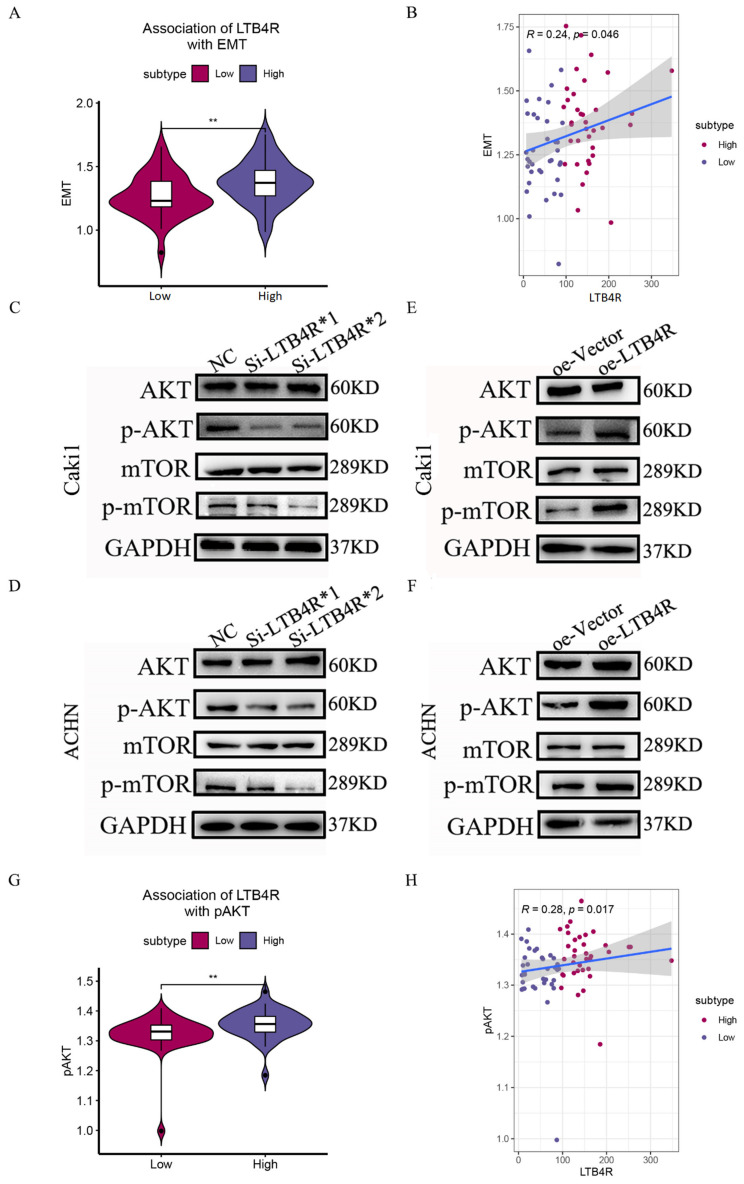
The association of LTB4R with EMT and the effects of LTB4R on the AKT/mTOR pathway. (**A**) The difference of EMT scores defined by using ssGSEA between LTB4R high expressed group and LTB4R low expressed group. Wilcoxon rank-sum test, ** *p* < 0.01. (**B**) The association of LTB4R expression with EMT score. (**C**,**D**) Western blot analysis showed that knockdown of LTB4R reduced the expression of p-AKT and p-mTOR in Caki1 and ACHN cells. (**E**,**F**) Western blot analysis showed that overexpression of LTB4R increased the expression of p-AKT and p-mTOR in Caki1 and ACHN cells. (**G**) The difference of AKT/mTOR signaling pathway scores defined by using ssGSEA between LTB4R high expressed group and LTB4R low expressed group. Wilcoxon rank-sum test, ** *p* < 0.01. (**H**) The association of LTB4R expression with AKT/mTOR signaling pathway score, (** *p* < 0.01).

We attempted to provide bioinformatic evidence regarding these relationships. We used two methods to analyze associations between LTB4R and related genes, including the comparison of differences by group samples according to LTB4R expression level and calculating the Pearson correlation. We found that ccRCC patients who had high LTB4R expressions also had higher expressions of BCL2 (Figure 7B, *p* < 0.05) and CDK4 levels (Figure 7F, *p* < 0.05) compared with ccRCC patients who had low LTB4R expressions. There was a trend for CCND1 (Figure 7D, *p* > 0.05) and CDK2 (Figure 7E, *p* > 0.05) levels to be higher in patients who had high LTB4R expressions than in patients who had low LTB4R expressions. This suggested positive correlations of LTB4R with these genes (BCL2: R = 0.23, *p* = 0.055; CCND1: R = 0.12, *p* = 0.32; CDK2: R = 0.15, *p* = 0.21; and CDK4: R = 0.25, *p* = 0.034). Patients who had high LTB4R expressions had lower BAX levels (Figure 7A, *p* < 0.05), and their CASP3 (Figure 7C, *p* = 0.2) tended to decrease. EMT scores were first calculated using single a sample gene set enrichment analysis (ssGSEA) via the R package “GSVA”. In addition, patients who had high LTB4R expressions had higher EMT levels (Figure 8A, *p* < 0.01). There was a positive correlation between LTB4R and EMT (Figure 8B, R = 0.24, *p* = 0.046).

### 3.9. LTB4R Regulation of the AKT/mTOR Signaling Pathway

PI3K/AKT/mTOR has been shown to be frequently activated in various cancer pathways and is an important target for cancer therapy [25]. Relevant studies have shown that phenstatin inhibited cell proliferation and induced apoptosis through the LTA4H–BLT1–AKT pathway, thereby inhibiting the occurrence of skin cancer. Therefore, we focused our attention on the AKT/mTOR pathway, a star pathway related to apoptosis and proliferation. Using Western blot experiments, we found that p-AKT and p-mTOR expression decreased in Caki1 and ACHN cells after LTB4R knockdown (Figure 8C,D). In contrast, p-AKT and p-mTOR expressions increased after LTB4R overexpression in ccRCC cells (Figure 8E,F). In addition, we attempted to validate the relationship between LTB4R and the AKT/mTOR pathway via bioinformatic analysis. The AKT/mTOR pathway score was first calculated using a single sample gene set enrichment analysis (ssGSEA) via the R package “GSVA” [26]. ccRCC patients who had high LTB4R expressions showed higher AKT/mTOR pathway scores (Figure 8G, *p* < 0.01). Correlation analysis indicated that LTB4R was positively associated with the AKT/mTOR pathway (Spearman’s R = 0.28, *p* = 0.017; see Figure 8H), which was consistent with our experimental results. These phenomena indicated that LTB4R regulated the AKT/mTOR pathway to affect the occurrence and development of ccRCC.

## 4. Discussion

Clear cell renal cell carcinoma (ccRCC) has a high degree of immune infiltration, but the impact of immune heterogeneity on ccRCC’s clinical outcome has not been fully characterized [27]. A recent study reported that the transcriptome analysis of human ovarian cancer peritonitis suggested that LTB4 and other arachidonic acid metabolites (PGE2 and PGI2) were associated with early cancer recurrence [28]. In addition, LTB4 can promote tumor growth by coordinating the pro-inflammatory microenvironment and directly affecting the proliferative capacity of tumor cells [29]. Many studies have reported that immune checkpoint blockade (ICB) therapy and combination regimens significantly improve survival in ccRCC patients [30,31]. Therefore, based on our previous studies [12], we further investigated LTB4R’s biological functions and mechanisms in the development of renal clear cell carcinoma.

In this study, we used bioinformatic analysis based on TCGA and GTEx databases to explore the relationship between LTB4R and ccRCC. We found that a high expression of LTB4R was related to a poorer prognosis and a more advanced ccRCC clinical stage. Next, via CCK8 assay, we found that LTB4R overexpression promoted ccRCC cells’ proliferation, whereas LTB4R knockdown inhibited ccRCC cells’ proliferation. Using scratch and transwell assays, we found that LTB4R overexpression promoted ccRCC cells’ migration and invasion, whereas LTB4R knockdown inhibited ccRCC cells’ migration and invasion. Flow cytometry analysis indicated that LTB4R could also inhibit apoptosis and promote the cell cycle of ccRCC cells. Furthermore, using protein blotting experiments, we confirmed that apoptosis-related proteins, BAX and c-CASP3, decreased, and BCL2 increased, after LTB4R overexpression, whereas BAX and c-CASP3 increased, and BCL2 decreased, after LTB4R knockdown. Cyclins CCND1, CDK2, and CDK4 increased after LTB4R overexpression, and decreased after LTB4R knockdown. In contrast, using a Western blot assay, we found that LTB4R promoted the EMT process in ccRCC cells. Bioinformatic analysis verified some of these results.

The AKT/mTOR signaling pathway can regulate cancer cell proliferation, growth, survival, and angiogenesis; its abnormal activation is also closely related to tumorigenesis and progression [32,33]. Related studies show that phenylbutazone inhibits cell proliferation and induces apoptosis through the LTA4H–BLT1–AKT pathway, thereby suppressing the development of skin cancer [34]. To verify the relationship between LTB4R and the AKT/mTOR signaling pathway in renal clear cell carcinoma, we performed a Western blot assay and found that LTB4R positively regulates the AKT/mTOR pathway, affecting the development of renal clear cell carcinoma.

In summary, our experimental results consistently suggest that high expression of LTB4R can be an independent risk factor for renal clear cell carcinoma. Functionally, LTB4R promotes the migration, invasion, proliferation, and apoptosis of renal cancer cells; mechanistically, LTB4R influences the progression of renal clear cell carcinoma by regulating the AKT/mTOR signaling pathway and, thus, the development of renal clear cell carcinoma. Therefore, LTB4R has promise as a novel prognostic biomarker for renal clear cell carcinoma. However, the most significant limitation of our study was that we did not validate LTB4R in vivo. We need to verify our results at the animal level. Furthermore, we will also more deeply explore the upstream and downstream mechanisms of LTB4R’s regulation of ccRCC development to obtain additional reliable evidence for the application of LTB4R in clinical targeted therapy. In addition, bioinformatic analysis indicated that some genes, such as BCL2, did not show significant correlation with LTB4R, perhaps because the public dataset was small. We will validate these correlations using our own data in the near future.

## 5. Conclusions

In conclusion, our study found that a high expression of LTB4R was associated with a poorer prognosis and a higher clinicopathological stage in ccRCC patients. We found that LTB4R promoted cell proliferation, migration, and invasion, as well as the development and progression of ccRCC by regulating the AKT/mTOR signaling pathway. Our results reveal that LTB4R is an important renal cancer biomarker and may be a highly specific therapeutic target. 

## Data Availability

The date presented in this study are available on request from the corresponding author upon reasonable request.
